# Understanding the roles of the microbiome in autoimmune rheumatic diseases

**DOI:** 10.2478/rir-2023-0027

**Published:** 2023-12-19

**Authors:** Abhimanyu Amarnani, Gregg J. Silverman

**Affiliations:** Department of Medicine, NYU Grossman School of Medicine, New York, NY USA

**Keywords:** microbiome, lupus, Ruminococcus gnavus, lupus flare, post intestinal-bloom, autoimmune syndrome

## Abstract

The gut microbiome represents a potential promising therapeutic target for autoimmune diseases. This review summarizes the current knowledge on the links between the gut microbiome and several autoimmune rheumatic diseases including rheumatoid arthritis (RA), systemic lupus erythematosus (SLE) spondyloarthropathies (SpA), Sjogren’s syndrome (SS), and systemic sclerosis (SSc). Evidence from studies of RA and SLE patients suggests that alterations in the gut microbiome composition and function contribute to disease development and progression through increased gut permeability, with microbes and microbial metabolites driving an excessive systemic activation of the immune system. Also, there is growing evidence that gut dysbiosis and subsequent immune cell activation may contribute to disease pathogenesis in SpA and SS. For SSc, there are fewer, but these are still informative, reports on alterations in the gut microbiome. In general, the complex interplay between the microbiome and the immune system is still not fully understood. Here we discuss the current knowledge of the link between the gut microbiome and autoimmune rheumatic diseases, highlighting potentially fertile areas for future research and make considerations on the potential benefits of strategies that restore gut microbiome homeostasis.

## Introduction

Autoimmune rheumatic diseases are a diverse and heterogeneous group of disorders that affect tens of millions of people worldwide ^[1]^ and are characterized by the immune activation with clonal expansions of lymphocytes that target the host’s own tissues and organs. While there has been an uneven improvement in outcomes for several of these autoimmune diseases, their etiology and pathogenesis are not yet fully understood. The microbiome, which includes the diverse collection of microorganisms that reside on and within the human body,^[2]^ has emerged in recent years as a potential player that can shape immune responses and contribute to the pathogenesis of various autoimmune diseases. This review focuses on emerging advancements that link the gut microbiota to immune dysregulation in rheumatoid arthritis (RA), systemic lupus erythematosus (SLE), spondyloarthropathies (SpA), Sjogren’s syndrome (SS), and systemic sclerosis (SSc).

The microbiome-host relationship represents a complex interplay between the host and its resident microbial communities whereby gut microbes play a crucial role in maintaining gut barrier integrity and immune homeostasis while preventing the expansion and mucosal invasion of potentially harmful species.^[3,4]^ While some studies have shown a correlation between gut microbiota imbalances (also termed dysbiosis) and inflammatory and autoimmune conditions,^[5]^ recent reports have suggested that the overgrowth, or bloom, of typically harmless gut bacteria can perturb the gut epithelial barrier and trigger immune responses to microbial antigens. This can lead to the onset or flare-up of autoimmune rheumatic diseases.^[6]^ In one example, the gut-joint hypothesis proposes that microbial dysregulation in the gut can lead to immune cell migration to joints, driving inflammatory responses.^[7]^

**Figure 1.**
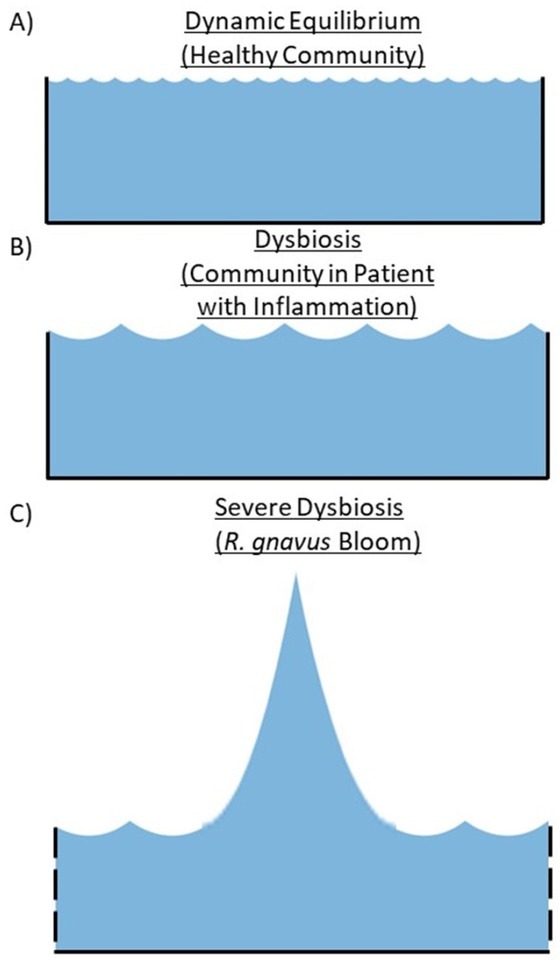
Conceptual Depiction of Dysbiosis and Microbiota Blooms Represented As Waves. A healthy gut microbiota includes a complex community with thousands or more distinct species that occupy anatomic and metabolic niches and a range of autoimmune diseases correlate with microbiota imbalances – also termed dysbiosis. In our studies, patients with lupus nephritis, especially the most severe cases, can develop expansions of single species and strains, also termed blooms. (A) A healthy gut microbiota community includes a dynamic equilibrium that is resilient following stressors such as nutritional and viral infections that generally reset to the preexisting balance. (B) Dysbiosis can be associated with inflammatory conditions and reflects shifts in the relative abundance of bacteria, reflecting metabolomic changes with some expansion of gut microbiota. (C) Severe dysbiosis can be associated with blooms that include large expansions of specific microbial species (e. g. R. gnavus in SLE) with a community diversity that is not resilient to stressors, though it is uncertain if blooms are causal in autoimmune diseases or reflect inflammation secondary to patients’ underlying systemic disease states. Increased gut epithelial permeability secondary to host inflammatory and/or microbial metabolomic changes also occur. These depictions are oversimplifications representing the (A) balance vs. B) dysbiotic imbalances that occur in many patients with inflammatory and autoimmune diseases C) blooms of individual potential pathobionts, in the highly complex communities that reside within the human intestine.

In patients with active RA and SLE, studies have shown that dysbiosis of gut microbiota community composition in association with altered metabolic pathways. Dysbiosis in the gut has also been associated with increased intestinal permeability in RA, SLE, SpA, and other autoimmune diseases, as discussed below.^[8,9]^ Specifically, it has been suggested, at least in some cases, that a translocation of bacterial products into the draining lymph nodes provides microbial exposure to the immune system for the exacerbation of pathologic inflammatory responses (Figure 1).^[10]^

To define a paradigm for the gut microbiome-host relationship, one can consider the billions of bacteria in the gut as an ocean-like ecosystem-many species residing within a specific niche, local microenvironments and microbiota subcommunities. The dynamic forces that govern the relative abundance of the multitude of diverse species are in part affected by the downstream flow of gut contents. The mucus layer generated by gut epithelial cells contributes to a functional barrier. High local levels of secreted dimeric IgA produced by mucosal plasma cells recognize certain species and may hasten their clearance, impede their proliferation, and decrease their interactions with intestinal epithelial cells. The gut barrier microenvironment and its adjacent mucosal layer function to rapidly mobilize cellular defenders to avoid infectious disease, targeting pathogens from crossing the barrier. When there is a physiologic balance between local commensal species, both pathogenic bacteria and otherwise commensal pathobionts are retained within the gut. Yet, the intestinal epithelial lining is not impenetrable. The expansions, or blooms, of select bacterial species- or local inflammatory responses-can increase intestinal permeability through receptor-mediated mechanisms affected by apical epithelial production of zonulin.^[8]^ Specifically, dysfunction in the intestinal barrier is reported to occur in the absence of substantial tissue injury by tight regulation of the zonulin pathway. Zonulin modulates the intracellular formation of tight junctions between intestinal epithelial cells lining the gastrointestinal (GI) tract, which play a crucial role in maintaining gut barrier integrity by impeding the passage of molecules from the intestinal lumen into draining lymphatics and subsequently into the bloodstream. In this setting, intestinal bacteria, and their inflammatory products, can traverse the mucus and intestinal epithelial barrier, favoring microbial-immune interactions. If host characteristics promote gut permeability, including through zonulin-mediated tight junction modifications that affect the gut epithelium barrier, then a lower amount of microbiota is sufficient to cross the gut epithelium. The increased gut permeability, along with intestinal cell death, can result in (auto) antigenic stimulation and enhanced immune signaling that can contribute to breaches in peripheral immune tolerance.

Herein, we begin by considering the historical context of the intersecting fields of microbiology and rheumatology, and then discuss the mechanistic links between commensal microbial dysbiosis and RA, SLE, SpA, SS and SSc. We will also discuss the proposed molecular mimicry mechanisms in this burgeoning field.

## A Historical Perspective

Autoimmune diseases now affect an estimated 5%–10% of the western populations.^[1,11]^ Though inherited genetic factors certainly contribute to susceptibility, environmental factors are also postulated to function as triggers for disease initiation and later flares. Gut microbiota communities, which consist of trillions of microorganisms residing in the GI tract,^[2]^ play essential roles in the production of vitamins, nutrients availability, and general immune regulation and homeostasis. Moreover, there is increasing recognition of the connection between infection and dysbiosis with gut microbiota communities in autoimmune pathogenesis.

In the earliest formulation of the theory of autoimmunity, which dates to the early 1900s, the French immunologist Paul Ehrlich proposed the concept of “horror autotoxicus”(fear of self-toxicity), which refers to the ability of the immune system to distinguish between self and non-self.^[12]^ Since then, steady progress has been made to understand the features and mechanisms responsible for autoimmune pathogenesis, including the molecular mechanisms by which the immune system balances receptor mediated processes that enable distinguishing self from non-self. Especially in the context of infectious disease, several studies have provided support for the molecular mimicry hypothesis, including those focused on rheumatic heart disease. Although controversies remain, the group A strain of the opportunistic commensal pathogenic bacterium, Streptococcus pyogenes, expresses a carbohydrate epitope, N-acetyl-beta-D-glucosamine (GlcNAc), to which patients with acute rheumatic fever make IgG2 antibodies, which in turn also target heart valve tissue, contributing to rheumatic heart disease.^[13,14]^ Similarly, a region within Epstein–Barr nuclear antigen 1 (EBNA1), an immunodominant protein of Epstein-Barr virus- a common human pathogen that in most cases does not cause clinical symptoms in infected individuals- was shown to have homology with an epitope within human myelin and could induce autoimmune encephalitis in a murine model system of multiple sclerosis.^[15]^

However, molecular mimicry may only represent a possibility. Another theory includes that certain bacteria and viruses contain proteins that can lead to activation of T cells or B cells and promote dysregulation.^[16, 17, 18]^ Enhanced immune signaling and inflammation,^[19]^ lymphocyte activation,^[20]^ polyclonal Ig secretion,^[21]^ and antigen receptor-mediated lymphocyte apoptosis,^[22]^ have all been observed in the presence of gut microbiota dysbiosis. The gut barrier integrity is also influenced by the gut microbiome that can modulate immune responses by producing metabolites, such as the small chain fatty acid butyrate,^[22]^ although direct mechanistic links have yet to be proven.

## Rheumatoid Arthritis

RA is a chronic systemic autoimmune disease characterized by synovial inflammation, cartilage destruction, and bone erosion.^[23]^ While it has long been known that the pathogenesis of RA involves the interaction between genetic and environmental factors, more recent mounting evidence has also shown an involvement of microbial dysbiosis.

Patients with RA exhibit an altered gut and oral microbiota composition and function, with a reduction in beneficial bacterial species and increased pro-inflammatory bacteria. Specifically, taxon-level analysis identified an expansion of rare taxa, Actinobacteria, with a decrease in abundant taxa in RA patients compared to controls, and found three genera, Collinsella, Eggerthella and Faecalibacterium, to be associated with RA.^[24]^ Additionally, RA patients with the HLA-DRB1 allele epitope, and especially those who become anti citrullinated protein antibody (ACPA) positive, are at-risk of RA. These individuals often have dysbiotic subgingival microbiomes associated with an increased abundance of the commensal, Porphyromonas gingivalis, compared to controls.^[25]^

Human observational studies and murine models have provided valuable insights into the gut-joint axis in RA.^[26]^ At disease onset, RA patients had increased intestinal microbes including *Prevotella copri* compared to healthy controls, and the expansion of these rare lineage intestinal microbes associated with an increase in the proinflammatory cytokine interleukin-17 (IL-17).^[27]^ These findings correlated with lowered diversity of Clostridia, Lachnospiraceae, and Bacteroides in the RA gut.^[27]^ Beyond the gut microbiome, the composition of oral microbiota communities was altered in patients with RA. Particularly, P. gingivalis has been considered as an extra-articular trigger for RA and other autoimmune diseases.^[28]^ Periodontal disease has long been known to be associated with an increased risk of RA,^[25]^ but it remains controversial whether this could be a cause or a consequence of RA disease. Specifically, RA patients with positive anti-citrullinated protein antigen (ACPA) antibodies exhibit a higher abundance of *P. gingivalis* in their oral microbiota, and *P. gingi-valis* expresses peptidylarginine deiminiase (PAD), which can convert arginine to citrulline in host proteins, generating citrullinated antigens.^[29]^ Aggregatibacter actinomycetemcomitans,^[30]^ which is also linked to periodontitis, is another oral bacterial microbe which shares the ability to generate citrullinated autoantigens. More recently, a human gut commensal called Subdoligranulum didolesgii, was implicated in RA pathogenesis. This putative pathobiont, identified with the use of RA patient serum for antibody cloning, was subsequently found in murine gut colonization models to cause synovitis and deposition of complement and antibodies, even in the absence of an experimental adjuvant trigger.^[31]^

SKG mice, that spontaneously develop arthritis, when rederived as germ-free were protected from developing experimental arthritis, while colonization of germ-free mice with segmented filamentous bacteria (SFB) developed inflammatory polyarthritis.^[32]^ Also, colonizing SKG mice in specific pathogen free (SPF) conditions with fresh human feces from RA patients with high abundance of Prevotellaceae promoted the development of inflammatory polyarthritis.^[33]^

Microbial effects may be relevant for understanding whether a RA patient responds to treatment with disease-modifying anti-rheumatic drugs (DMARDs). For example, there is evidence that gut dysbiosis correlates with worse clinical responses to treatment with the most commonly used DMARD, methotrexate.^[34,35]^ Moreover, the treatment of RA with a tumor necrosis factor (TNF)-a inhibitor has been reported to also partially restore the balance within a patient’s gut commensal community, which supports the notion that systemic inflammation in RA may itself be a driver for gut dysbiosis.^[36]^

In RA, it has also been proposed that there may be clinical benefits that can be attained by targeting gut dysbiosis through probiotic supplementation that introduces certain gut species. Monotherapy with ingestion of pathobiont Tripterygium wilfordii Hook F has been demonstrated to be non-inferior to methotrexate monotherapy in controlling disease activity in a small, although non-blinded, study of DMARD-naïve rheumatoid arthritis patients.^[37]^ Additionally, clinical trials that assessed the use of probiotics in RA patients found that probiotic supplementation (with Lactobacillus acidophilus, Lactobacillus casei, and Bifidobacterium bifidum) can improve clinical symptoms and markers of inflammation.^[38,39]^ Probiotic supplementation also significantly decreased insulin resistance and improved lipid metabolism in RA patients.^[38]^ However, the use of probiotics in RA needs further validation before it can be adopted for general use.

Another approach with potential benefits is the use of short-chain fatty acids (SCFAs), which are metabolites produced by gut bacteria during fermentation of dietary fiber that is otherwise indigestible. In patients with RA, gut dysbiosis can alter the composition of gut microbiota, leading to changes in the production of SCFAs. SCFA levels may themselves play a role in the pathogenesis of RA by directly modulating immune cell function and associated inflammatory responses. The most prominent SCFAs are acetate, propionate, and butyrate, which have postulated immunomodulatory effects in RA patients.

SCFAs have been reported to inhibit the onset of experimental arthritis, and serum butyrate levels were found to be depressed at times before the onset of arthritis.^[40]^ Conversely, in mice the administration of SCFAs improves the severity of arthritis, in part through the regulation of B cell differentiation mediated by free fatty acif type 2 receptor (FFA2), a G protein coupled receptor for fatty acids.^[41]^ In a prospective cohort study of individuals with an increased risk for developing RA, the subjects who progressed to clinical arthritis had lower serum levels of total SCFAs, and especially butyrate, and acetate, at baseline compared to those who did not progress to overt disease.^[42]^ These early findings support the need for further investigations on the role of SCFAs in RA. Other studies of dietary interventions in RA patients showed that a low-calorie, Mediterranean diet significantly changed the metabolic pathways in the GI tract of RA patients, including the metabolism of amino acids and lipids with associated microbiome differences between RA responders and non-responders to treatment.^[43,44]^

The above-described studies suggest that future therapeutic considerations for RA may include dietary modification, and the direct targeting of microbial dysbiosis, which by themselves have limited potency, but which might be utilized in conjunction with conventional RA treatments. However, development of the most effective methodology to target dysbiosis will require further investigation.

## Systemic Lupus Erythematosus, murine models and autoantigens

Murine lupus models have suggested the potential relevance of certain mechanisms triggered by gut dysbiosis. Mice gut colonized with segmented filamentous bacteria displayed worsening of lupus nephritis which was associated with increased CD206^+^ macrophage infiltration.^[45]^ Enterococcus gallinarum, a human gut commensal bacterium, when introduced into the mouse GI tract of germ-free mice induced anti-dsDNA antibodies. Of note, E. gallinarum translocation was documented, as this microbe was be cultured in these colonized mice from blood, lymph fluid, and liver.^[46,47]^

Several studies have suggested that bacterial antigens and metabolites can induce production of autoantibodies in SLE patients. These may include DNA-binding amyloids (curli),^[48]^ bacterial lipopolysaccharides, lipoglycans, teichoic acids, and other bacterial products that can activate the innate immune system via toll-like receptors (TLR) and other pattern-recognition receptors on immune cells.^[49]^ Furthermore, microbe-immune cell interactions may trigger neutrophils to undergo cell death. One such pathway may result in release of neutrophil extracellular traps, as neutrophiles undergo NETosis that enhance immune recognition of commensal antigens that represent orthologs of self-antigens. In one compelling example, Ro60, which is an RNA binding protein that is a common target of autoantibody responses in patients with lupus and Sjogren’s syndrome, was shown to also become recognized by specific T cells in lupus-prone mice colonized with commensal bacteria that naturally express Ro60 orthologs.^[50,51]^ Notably, depletion of Ro60 ortholog-expressing bacteria reduced the *in vivo* representation of activated Ro60-specific T cells.^[50]^

## Studies of the gut microbiome in cohorts of SLE patients

A number of independent cross-sectional (i. e., single timepoint) reports have documented gut microbiota dysbiosis in patients with SLE.^[3,44,46,47,52, 53, 54, 55, 56, 57, 58]^ While the gut microbiome in health is dominated by anaerobic species, SLE patients have a decrease in anaerobes, Firmicutes and Bacteroidetes, and an increase in Proteobacteria aerobic species that may better compete in an inflamed host.^[54]^ Studies of fecal samples from SLE patients with active disease have also shown lower diversity and richness compared to healthy controls.^[54]^ Notably, Azzouz *et al*. documented a decrease in microbiota alpha-diversity was inversely correlated with disease severity as measured by the SLE disease activity index (SLEDAI).^[52]^ Impaired intestinal barrier function and associated translocation of microbiota and metabolic products has provided independent evidence that imbalances in the gut microbiota may contribute to SLE disease pathogenesis.^[6,59]^ Independent reports on cohorts from diverse geographic areas have implicated expansions of the obligate anaerobic commensal, *Ruminococcus (blautia) gnavus* of the family Lachnospiraceae, in patients with active SLE.^[52,60]^ In a cross-sectional study of a cohort in New York City, compared to low stable gut abundance of *R. gnavus* at a mean 0.1 %, *R. gna-vus* was present at five-fold higher levels in SLE patients, and abundance correlated with the level of lupus disease activity. Highest levels were documented in patients with active renal involvement (i. e., lupus nephritis). Notably, patients with active lupus nephritis displayed the highest levels of serum IgG antibodies to a *R. gnavus* strain-specific antigen, which represents a cell wall-associated lipoglycan ^[52,61]^ supporting that gut leak of this antigen is present in affected lupus patients.

In the largest cross-sectional study described to date, in a report from Peking University, examinations of 117 untreated SLE patients documented that dysbiosis was common and that, expansions of *R. gnavus* were the most distinct feature identifying patients with active lupus nephritis.^[53,62]^ Serum antibody responses to the lipoglycan produced by some *R. gnavus* strains have also been correlated with active lupus nephritis in a cohort that included patients with new onset SLE studied at the Karolinska Institute in Sweden.^[61]^ Taken together, these studies indicate that *R. gnavus* expansions arise in active lupus patients on three continents.

To directly investigate the pathogenic potential of *R. gna-vus* gut colonization, a number of strains have been isolated from fecal samples from patients with active lupus nephritis.^[63]^ Analysis of the genome of these strains documented the presence of a number of genes believed to absent in strains from healthy individuals, but which adapt the strain for survival and imbue ecological competitive advantages in a host with systemic inflammation.^[64]^ Gut colonization with strains from lupus patients induced dramatically increased gut permeability, and induced systemic production of anti-lipoglycan antibodies as well as anti-DNA autoantibodies,^[60]^ but this did not occur following colonization with a strain isolated from a healthy individual. While anti-lipoglycan antibodies naturally arise in the absence of previous immunization, which therefore may be considered a form of natural antibodies,^[63]^ the pathogenic potential of some clones of anti-DNA antibodies has been well established.^[65]^ Future studies will be needed to determine if the novel lipoglycan produced by some *R. gnavus* strains can be utilized as a biomarker for lupus nlupus (LN) disease.

In the first report of a longitudinal study of patients with SLE, fundamental abnormalities in the stability of gut communities in these patients were documented.^[64]^ In health, the composition of the gut microbiota community is typically stable over time, representing a dynamic equilibrium that is maintained by currently uncertain mechanisms, In contrast, the overall composition of the gut microbiota in patients with SLE were unstable, and drifted over time, which may indicate that they are particularly vulnerable to disruptions caused by insults such as intercurrent minor infections, food additives in processed food, or perhaps even antibiotics.^[64]^ Hence, the gut microbiome in SLE patients appears to lack resilience and generally unable to return to a pre-insult state,^[66]^ favoring the dysbiosis of the gut microbiome.^[64]^ It is therefore speculated that this can lead to increased gut permeability and secondary systemic inflammation that may stoke and worsen self-perpetuating pathways of autoimmune pathogenesis (Figure 1).

These longitudinal studies also demonstrated in more than 40% of a small cohort, flares of lupus nephritis occurred at time of blooms of *R. gnavus*, which were 20–90 fold greater than the gut abundance in healthy individuals. While ephemeral blooms of two other anaerobic commensal species were also documented, these other species were not associated with disease flares. Overall, these studies suggest that dysbiosis of the gut microbiota, associated with unstable gut communities, and blooms of lipoglycan-associated *R. gna-vus* strains, may be associated with breaches of immune tolerance, promotion of autoantibody production, and autoimmune renal disease flares.

## Spondyloarthropathies

Spondyloarthropathies (SpA) include ankylosing spondylitis (AS), psoriatic arthritis (PsA), reactive arthritis, and non-radiographic SpA.^[67]^ Despite considerable progress in understanding the pathogenesis of SpA, the exact etiology driving initial disease development and subsequent flares remains unknown. Recent evidence suggests that dysbiosis of the gut microbiome may be a contributing player.^[68]^

In AS and PsA, distinct patterns of gut microbiota dysbiosis have been identified, with an increased abundance of bacterial taxa Prevotella and Klebsiella, representing Proteobacteria and Actinobacteria.^[69, 70, 71]^ As in SLE and RA, increased gut permeability correlating with elevated levels of zonulin and increased intestinal permeability have been identified in patients with AS.^[72]^ Murine models suggest that the effect of gut dysbiosis on zonulin mediated regulation of intestinal permeability is more pronounced in females.^[60]^ This unexpected finding could in part explain why many autoimmune diseases, including RA and SLE, predominantly affect women.

As for AS, rats made transgenic for the human antigen presentation gene, HLAB27, developed arthritis, characteristic spinal as well as skin and nail changes, and bowel involvement however, when these rats rederived in a germ-free environment, disease did not develop.^[73, 74, 75]^ In patients with AS, ileal biopsies showed an increased expression of zonulin, compared to control patients.^[68]^ Though the associated mechanisms have not been well defined, HLAB27 clearly is a disease susceptibility factor, and altered gut permeability appears to be involved in pathogenesis. Notably, in one report, patients with Crohn’s disease and the extraintestinal manifestation of SpA had significant gut expansions of *R. gnavus* compared to healthy controls.^[76]^

## Sjogren’s Syndrome

Recent studies in patients with SS have demonstrated that the oral microbiome,^[77]^ as well as the gut microbiome,^[78]^ significantly differ from that in healthy individuals. Further, in SS patients, correlative evidence suggests a pathogenic influence of certain microbial species within the gut, ocular, and oral microbiota communities, which highlights the now termed gut-ocular-oral axis.^[77]^ SS patients are also reported to have an increase abundance of Actinomyces and Lactobacillus in both stool and oral samples,^[79]^ which implicated these taxa as potential pathobionts. This suggests the possibility that oral bacteria or their products may traverse buccal epithelial cells through local defects in the epithelial barrier. Furthermore, T cell epitope mimicry between SS antigen A (SSA) /Ro60 and various bacteria have been postulated as disease drivers.^[80]^ In an independent cross-sectional study of human SS saliva, based on 16S rRNA amplimer sequencing taxonomic distribution in these communities were documented. These studies have identified four genera of bacteria, Bifidobacterium, Lactobacillus, Dialister and Leptotrichia as significantly different in abundance in SS patients compared to healthy controls.^[81]^

## Systemic Sclerosis

There is also emerging evidence of gut microbial dysbiosis in patients with SSc.^[82,83]^ In cross-sectional studies of gut microbiome communities in patients with SSc, relatively subtle differences in the microbial diversity have been described. As in SS, there were no significant differences in community alpha-diversity in SSc and healthy control subjects. However, on subgroup analysis a decrease in alpha diversity was found in SSc patients with GI symptoms as compared to those without GI symptoms and healthy controls.^[84]^ An increase in gut abundance of the aerobic species, Escherichia coli, and a decrease in gut abundance of beneficial bacteria, such as Bifidobacterium and Lactobacillus, have been observed in SSc patients.^[85,86]^ In a small double-blinded, placebo-controlled trial, SSc patients that received a predetermined combination of probiotics had increased stool microbiota alpha diversity and had symptom improvement, specifically for GI reflux, although overall gastrointestinal tract symptom questionnaires were not significant difference compared to placebo.^[87]^ Though early stage, these encouraging results suggest that further mechanistic studies are merited.

## Perspective

Advances in the still young field of microbiome research have shed light on the intricate relationships between the gut microbiota and rheumatic diseases. Research progress has provided new avenues for understanding the pathogenesis and treatment options for these complex conditions that are associated with great morbidity and disability, and at times early mortality. The studies summarized in this review suggest there is a significant impact of microbial dysbiosis on disease development and progression. However, future research must emphasize longitudinal studies that simultaneously track changes in the microbiome and immune system over time with repeat sampling, investigate microbial metabolites produced in the gut and their affect immune tolerance, as well as address how medications and diet can alter the composition of gut microbial species in these patients. Beyond this, microbial network analysis could further characterize the networks of species that dynamically shift during the course of disease. Such studies will better define the specific roles of many different individual candidate pathobionts.

These studies suggest that in some patients, specific microbial species, associated with disease activity and autoantibody production, have identifiable antigens that can serve as valuable diagnostic and prognostic tools. Further investigations are needed to elucidate the mechanisms by which gut microbiota influence rheumatic diseases, including the potential involvement of gut barrier dysfunction, immune dysregulation, and molecular mimicry. Looking ahead, personalized microbiome-based therapies for targeted interventions may be utilized to restore microbial resilience and thereby also promote immune balance in patients with rheumatic diseases. By harnessing the power of the gut microbiota, future research endeavors can revolutionize our understanding and treatment of rheumatic diseases.

## Conclusion

As a resource for the reader, we have highlighted example key reports on the microbiome in patients with rheumatic autoimmune diseases (Table 1), with apologies to the authors whose work we have not included. Intervention studies are currently in progress while others are still in the planning stages, with an overarching goal of understanding whether normalization of gut dysbiosis by itself, or through the targeting of specific bacterial strains, can ameliorate symptoms and/or improve disease manifestations. Such therapies may need to be personalized and tailored according to microbiome of individual patients, which we now know is affected by diet, and genetic background.

**Table 1.** Recent reports on microbial dysbiosis in patients with rheumatic autoimmune disease

Reference	Year	Disease Studied	Species	Summary
[36]	2020	Rheumatoid Arthritis	Human	Investigates the impact of the Mediterranean diet on disease activity and gut microbiota in RA patients.
[37]	2018	Rheumatoid Arthritis	Human	Evidence that Tripterygium wilfordii Hook F affects responses in active RA patients to methotrexate treatment.
[31]	2022	Rheumatoid Arthritis	Human	Identification of an arthritogenic strain of Subdoligranulum that is associated with clonal IgA and IgG autoantibodies in at-risk individuals.
[38]	2017	Rheumatoid Arthritis	Human	Examines the clinical and metabolic response to probiotic supplementation in patients with rheumatoid arthritis in a randomized, double-blind, placebo-controlled trial
[24]	2016	Rheumatoid Arthritis	Human	Rheumatoid arthritis patients show an expansion of rare strains of intestinal species, including Eggerthella, Collinsella, and Faecalibacterium, that potentially contribute to disease pathogenesis
[25]	2021	Rheumatoid Arthritis	Human	Oral microbiome dysbiosis, including increased levels of Prevotella and Veillonella, is observed in anti-CCP positive individuals at risk for rheumatoid arthritis.
[27]	2013	Rheumatoid Arthritis	Human	Expansion of intestinal Prevotella copri is associated with increased susceptibility to arthritis
[40]	2020	Rheumatoid Arthritis	Human and mouse	Inhibition of zonulin and improvement of intestinal epithelial barrier function may help prevent onset of RA.
[35]	2021	Rheumatoid Arthritis	Human and Mouse	Methotrexate broadly alters the human gut microbiota, with varying sensitivity across strains. In rheumatoid arthritis patients, MTX impacts gut bacterial taxa and gene family abundance differently in responders and non-responders, ultimately affecting immune function.
[41]	2022	Rheumatoid Arthritis	Mouse	Short-chain fatty acids (SCFAs) modulate B cell differentiation through the FFA2 receptor, leading to reduced inflammation and amelioration of RA signs and symptoms
[38]	2017	Rheumatoid Arthritis	Human	Examines the clinical and metabolic response to probiotic supplementation in patients with rheumatoid arthritis in a randomized, double-blind, placebo-controlled trial
[60]	2022	Lupus	Human	Evidence of sex-linked induction of zonulin-mediated intestinal permeability and autoimmunity by strains of Ruminococcus Blautia gnavus isolated from patients with active lupus nephritis.
[50]	2018	Lupus	Human, Mouse	Evidence that commensal orthologs of the human autoantigen Ro60 function as triggers of autoimmunity in lupus.
[54]	2014	Lupus	Human	Investigates intestinal dysbiosis associated with systemic lupus erythematosus in remission.
[52]	2019	Lupus	Mouse and Human	Lupus nephritis is disease-activity directly correlated with reduced alpha diversity and expansions of the gut commensal, Ruminococcus blautia gnavus, with associated host immune responses
[72]	2015	Ankylosing Spon- dylitis	Human	Evidence that dysbiosis and zonulin upregulation in ankylosing spondylitis patients with impaired gut barrier function.
[69]	2015	Psoriatic Arthritis	Human	Patients with psoriatic arthritis exhibit decreased bacterial diversity in the gut microbiota, resembling dysbiosis observed in patients with inflammatory bowel disease.
[76]	2023	Ankylosing Spondylitis	Human	Both disease activity and HLA-B27 status contribute to gut microbiome dysbiosis in spondyloarthritis patients, with alterations in abundance of Bacteroides, Faecalibacterium, and Dialister, as well as Ruminococcus gnavus
[88]	2022	Inflammatory Bowel Disease	Human, Mouse	Evidence that the systemic anti-microbiota IgG clonal expansions recognize gut bacteria that translocate across the gut barrier.
[80]	2016	Sjögren’s Syndrome	Human	Explores T cell epitope mimicry between Sjögren’s syndrome antigen A (SSA) /Ro60 and various bacteria.
[79]	2023	Sjogren’s syndrome	Human	Treatment-naïve patients with primary Sjögren’s syndrome exhibit compositional and functional aberrations in their gut microbiota, including Lactobacillus salivarius, Bacteroides fragilis, Ruminococcus gnavus. Lactobacillus salivarius was the most discriminating species.
[86]	2020	Systemic Sclerosis	Human	Explores the association of gut microbiome with disease severity and clinical outcomes in systemic sclerosis.
[88]	2022	Inflammatory Bowel Disease	Human, Mouse	Evidence that the systemic anti-microbiota IgG clonal expansions recognize gut bacteria that translocate across the gut barrier.
[64]	2023	Lupus	Human	Longitudinal analyses revealed inherent instability and transient blooms of Ruminococcus (blautia) gnavus (RG) in the gut microbiota of SLE patients, particularly during LN flares, with RG strains expressing a novel immunogenic lipoglycan

Cross-sectional studies have been a good starting point but have limitations as such study methods cannot elucidate the time-dependent relationships between gut microbiome and an individual’s host immune system. Future longitudinal studies can assess the dynamic changes of multiple species over time and evaluate the relative resilience of the overall microbiota ecosystem within the patients’ gut as compared to healthy individuals. Candidate microbial pathobiont species may also associate with disease-associated changes within microbiota communities. Such changes can be conceptualized as perturbations in a complex pool of microbial organisms, in which the most severe alterations are associated with blooms of individual pathobionts (Figure 1). Such shifts are also associated with functional breaches of the gut barrier that can induce inflammation that favor the competitive expansion of certain species and strains, including those implicated in pathogenesis, representing a feed-forward influence on autoimmune pathogenesis. A better understanding of the complex interplay of these altered communities with the host immune system can lead to strategies of intervention that are, at present, still in their infancy, yet, as indicated in small animal studies, have great potential.

In conclusion, while there is compelling evidence for the influence of altered microbiota communities in autoimmune rheumatic diseases, more research is still needed to further explore microbial blooms and the specific actors in the breach of the gut epithelial integrity, to ultimately develop new potential therapeutic targets.
